# Entropy generation from convective–radiative moving exponential porous fins with variable thermal conductivity and internal heat generations

**DOI:** 10.1038/s41598-022-05507-1

**Published:** 2022-02-02

**Authors:** Zia Ud Din, Amir Ali, Manuel De la Sen, Gul Zaman

**Affiliations:** 1 Department of Mathematics, University of Malakand, Khyber Pakhtunkhwa, Pakistan; 2 Department of Electricity and Electronics, Institute of Research and Development of Processes Faculty of Science and Technology, University of the Basque Country Campus of Leioa, 48940 Leioa, Spain

**Keywords:** Energy science and technology, Engineering, Mathematics and computing

## Abstract

The performance and thermal properties of convective–radiative rectangular and moving exponential porous fins with variable thermal conductivity together with internal heat generation are investigated. The second law of thermodynamics is used to investigate entropy generation in the proposed fins. The model is numerically solved using shooting technique. It is observed that the entropy generation depends on porosity parameter, temperature ratio, temperature distribution, thermal conductivity and fins structure. It is noted that entropy generation for a decay exponential fin is higher than that of a rectangular fin which is greater than that of a growing exponential fin. Moreover, entropy generation decreases as thermal conductivity increases. The results also reveal that entropy generation is maximum at the fin’s base and the average entropy production depends on porosity parameters and temperature ratio. It is further reveal that the temperature ratio has a smaller amount of influence on entropy as compared to porosity parameter. It is concluded that when the temperature ratio is increases from 1.1 to 1.9, the entropy generation number is also increase by $30\%$ approximately. However, increasing porosity from 1 to 80 gives 14-fold increase in average entropy generation.

## Introduction

In mechanical engineering, heat transfer is an extremely well-known phenomenon for different objects. If heat transfer rate is less than what is necessary, one of the best ways to boost heat transmission is to use an extended surface known as a fin. The mechanism of heat transfer through the fin is to conduct heat from the system to the outward surface of the fin through conduction and then transfer this heat to the circumferential medium through radiation and convection. Fins come in a variety of shapes, depending on the nature and use of the designs. Fin material and surrounding fluid play a vital role in temperature distribution. The requirement for improved heat exchangers has significantly increased in the design of industrial and electronic components, as the power used by computer micro-processors increased to $100\%$ in approximately two decades^1^.

The temperature distribution through fins have been extensively studied using various geometries. The heat flow, heat transfer coefficient, weight of star-shaped and annular fins were studied and compared concluding that Star-shaped fins perform better than annular fins^2^. Numerical and experimental investigations on W-type fin arrays revealed that the cooling influence of the W-type finned heat sink is significantly greater than that of the longitudinal parallel plate fin. Further, the Y-shaped fin has been studied and concluded that it is more useful in a cavity^3^. The porous fin of the T-shape has been analyzed, and it has been discovered that temperature distribution increases with the increase of porosity parameter and drops with increasing Biot number^4^. The applications of more porous fins to promote heat flow has been studied and revealed to be highly dependent on thermal conductivity, where the fin length has shown to be a function of the Rayleigh number^6^. The heat transfer from a trapezoidal structure’s longitudinal fin arrangement with common illumination has been studied in Ref.^5^. The numerical analysis of longitudinal porous fins of parabolic, rectangular and trapezoidal structures has presented in Ref.^7^. The heat exchange features of a steady magneto-hydrodynamic (MHD) flow of sheared thickening fluid were addressed in the presence of convective boundary conditions^10^. The heat exchange and flow properties of a copper-aluminum/water hybrid nanofluid in the existence of viscous dissolution (MHD), as well as the impact of the porous medium across a shrinking sheet, were investigated^11^. Similarly, the flow of second-order slip in a nanofluid through a moving thin needle has extensively studied^12^.

Several numerical techniques have been used to investigate heat transfer through convective fins having variable thermal conductivity. The finite difference method (FDM) and the Taylor transformation have been proposed to investigate heat transfer from an annular fin. The author investigated the influence of heat exchange from fin’s tip to the neighbouring fluid and emissivity. The results revealed that the Taylor transformation method has significant tools for evaluation of the second-order non-linear fins model^8^. The Adomian decomposition method (ADM) was applied to investigate a radiative-convective longitudinal fin with varying thermal conductivity^9^. The homotopy perturbation method (HPM) was used to study the effect of variable thermal conductivity on the thermal stress of the annular fin^13^. It has been determined that the heat exchange rate is affected by both thermo-geometric and the thermal conductivity parameters of the fin. The Homotopy analysis method (HAM) has applied to analyze coupled differential equations and obtain the competence of the convective longitudinal fin having variable thermal conductivity^14^. To optimize the structure and mass of a fin, the variational calculus approach has been used^15^. The decomposition technique was used to estimate the ideal length and affectiveness of a rectangular fin^16^. Similarly, a hybrid technique combining DTM and FDM has been used to examine fins having annular geometry^17^. The authors primarily investigates the impact of emissivity, heat transfer coefficient and absorptivity on temperature profile.

The generation of entropy and the temperature have a strong relationship. The strength of the random motion of particles is determined by temperature, while entropy is a measure of atomic disorder in a body. In engineering systems, thermodynamic optimization examine the function of entropy which explicitly answers the question of entropy generation^18^. In thermodynamic optimization, the rate of entropy production has become the objective function. Entropy generation minimization (EGM) is the subject of the modern era. Some basic engineering systems, such as thermal power station units, solar collectors, heat exchangers, air-conditioning systems, and others, could benefit from the EGM technique^19,20^. The use of EGM improves the combined effect of heat resistance and exchanger contact with the ambient fluid flow. The nature of the micro-channel is influenced by pressure drop and thermal resistance, according to previous research. EGM, on the other hand, suggested that the entropy generation rate be reduced as well. EGM is a technique of determining the best geometry and functionality. A perforated fin was improved using the EGM method^21^. According to the author’s calculations, the fin’s outer surface should be solid, while the inner side should be perforated. As a result, air movement across the fin was impossible, and the porous structure simply influenced the fin’s weight.

Engineers are working to improve thermal interaction and thermodynamic performance through the design of heat exchangers. As a result, a better heat exchanger provides the least amount of entropy. By studying entropy generation, a method for finding the ideal fin design and reducing energy loss in a thermal system has been developed^22^. Entropy production in nanofluids has also been studied^23^. For example, in a cuboid container filled with nanofluids, entropy formation and two-dimensional natural convection were examined concluded that the Rayleigh number and the solid volume fraction have direct and inverse relations with entropy generation^24^. The use of numerous entropy generating units as a main factor in defining heat exchanger efficiency was suggested^25^. In that technique, the overall generation of entropy associated to fluid friction and heat transmission was formulated. Because entropy production is proportional to the amount of energy lost, the study has expanded to include mass transfer and manufacturing costs^26^. Some studies has also attempted at the rates of local entropy formation in mixed convective flow over a transverse fins array at the vertical channel input^27^.

### Problem statement

The primary objective of this study is to analyze the thermal efficiency and entropy production in a convective–radiative exponential perforated fin with internal heat source based on convective heat transfer coefficient, surface emissivity and variable thermal conductivity.

## Mathematical formulation

**Figure 1:**
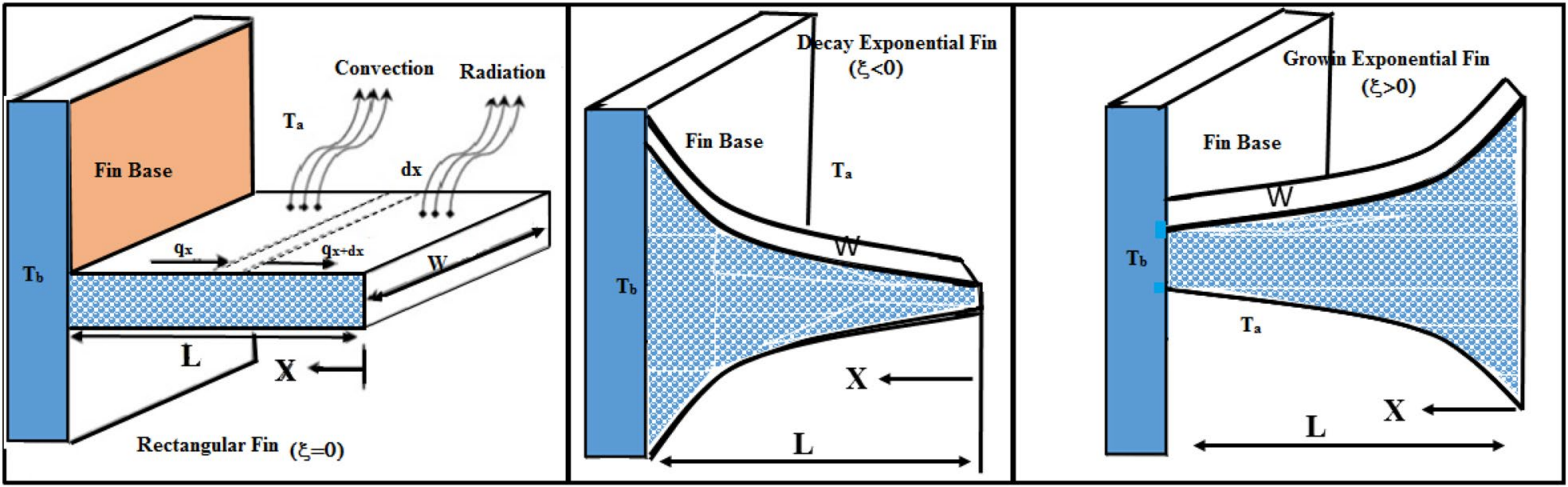
Schematic diagram of rectangular fin, decay and growing exponential fin.

Moving exponential perforated fin with and variable thermal conductivity and internal heat generation is considered. The dimensions of the fin are as follows: fin width *W*, length *L* and thickness *t* as presented in Fig. 1. The porous design allows the flow to move across the fin. The hot outside surface of the fin loses heat both by radiation and convection. If just radiation exists, or if induced convection is missing or weak, radiation would play an important part. To obtain the governing equation, it is supposed that the fin is isotropic, homogeneous and saturated with single-phase fluid, and the Darcy model is used to investigate the fluid-porous medium interaction.Furthermore, it is considered that both the solid and fluid bodies are in thermal balance with one another. The exponential fin shape is given by^28^

1 $$\begin{aligned} f(x)=\tau _{b}\,e^{\xi ^* x}, \end{aligned}$$

where $\xi ^*$ denote fin shape parameter^29^. It should be noted that $\xi ^* = 0$ indicate rectangular fin and $|\xi ^*| \ne 0$ represent exponential fin, while $\tau _{b}$ is the semi-fin thickness. Without loss of assumption, the thickness at the base is fixed to 1.

Based on Darcy’s model and the aforementioned assumptions the energy equation for porous fin can be expressed as

2 $$\begin{aligned} {\dot{q}}_{x}-\Big ({\dot{q}}_{x}+\frac{\partial q}{\partial x}dx \Big ) -{\dot{m}}C_{p}\Big [T-T_{a}\Big ]\,dx - h (1-\varphi )P \Big [T-T_{a}\Big ]dx + U \rho C_{p} f(x)\frac{d T}{d x}dx + {\dot{q}}f(x) -\varepsilon \sigma P \Big [T^4-T_{a}^4\Big ]dx = 0, \end{aligned}$$

considering $dx \rightarrow 0$ and simplifying

3 $$\begin{aligned} -\frac{d {\dot{q}}}{d x} -{\dot{m}}\, C_{p}\Big [T-T_{a}\Big ]-h\,(1-\varphi )\, P\,\Big [T-T_{a}\Big ]+ U\, \rho \, C_{p} \, f(x) \frac{d T}{d x} + {\dot{q}}\,f(x) -\varepsilon \, \sigma \, P \,\Big [T^4-T_{a}^4\Big ]=0. \end{aligned}$$

From Fourier’s law of heat conduction^30^, we have

4 $$\begin{aligned} q = - k_{eff}(T)\, f(x)\,A\,\frac{d T}{d x}. \end{aligned}$$

Putting Eq. (4) in Eq. (3), we obtain

5 $$\begin{aligned} \frac{d}{d x}\Big [k_{eff}(T)\, f(x)\,A\,\frac{d T}{d x} \Big ] -{\dot{m}}\, C_{p}\Big [T-T_{a}\Big ]-h\,(1-\varphi )\, P\,\Big [T-T_{a}\Big ]+ U\, \rho \, C_{p} \,f(x) \frac{d T}{d x} + {\dot{q}}\,f(x) - \varepsilon \, \sigma \, P \Big [T^4-T_{a}^4\Big ]=0. \end{aligned}$$

The rate of mass flow ${\dot{m}}$ of the air traveling across the permeable material is represented by^31^

6 $$\begin{aligned} {\dot{m}} = \rho \,V(x)\,W\,\triangle x, \end{aligned}$$

where *V*(*x*) represents buoyancy flow velocity at any point *x* can be achieved from Darcy’s law^32^ as

7 $$\begin{aligned} V(x)=\frac{\beta \,g\, K }{\nu }\Bigg [T-T_{a}\Bigg ]. \end{aligned}$$

The $k_{eff}(T)$ is variable thermal conductivity of the fin is defined as

8 $$\begin{aligned} k_{eff}(T) = \varphi \,k_{f}+(1-\varphi )\,k_{s}= k_{eff,a}\,\Big [1 + \lambda \,\Big (T - T_{a}\Big )\Big ]. \end{aligned}$$

Substituting Eqs. (6)–(8) in Eq. (5), we obtain

9 $$\begin{aligned}&\frac{d}{dx}\Bigg [(1 + \lambda (T - T_{a}) f(x)\,\frac{d T}{d x}\Bigg ] + \frac{U \,\rho \, C_{p}}{A\,k_{eff,a}}\, f(x) \,\frac{d T}{d x} -\frac{h\,(1-\varphi )\, P}{A\,k_{eff,a}}\Big [T-T_{a}\Big ] -\frac{\rho \,\beta \,g \, C_{p}\, K\, W}{A\,\nu \, k_{eff,a}}\Big [T-T_{a}\Big ]^2 +{\dot{q}}\,f(x)\nonumber \\&\quad -\frac{\varepsilon \, \sigma \,P }{A\, k_{eff,a}}\,\Big [T^4 - T_{a}^4\Big ]=0. \end{aligned}$$

To non-dimensionalize the above equation, we assume

$$\begin{aligned} \xi ^*=\frac{\xi }{L}, \, X=\frac{x}{L},\, \theta =\frac{T-T_{a}}{T_{b}-T_{a}},\, S_{h}=\frac{D_{a}\, R_{a}\,(L/t)^2}{k_{r}}, \, N_{c}^2 = \frac{h\,(1-\varphi )\, P\, L^2}{A\, k_{eff,a}}, \, N_{r} = \frac{\varepsilon \,\sigma \,P\, L^2 T_{b}^3}{A\,k_{eff,a}}, {\ddot{Q}} = \frac{{\dot{q}}\,L^2}{T_{b}k_{eff,a}}, \, P_{e}=\frac{U\, P\, L}{A\, \alpha }, \end{aligned}$$

simplifying the formulation of the moving exponential fin having variable thermal conductivity reduce to

10 $$\begin{aligned} \Big [1+\lambda \cdot \theta \Big ] \, \frac{d^2 \theta }{d X^2} + \Big [\xi \,(1+\lambda \cdot \theta ) + P_{e}\Big ] \, \frac{d \theta }{d X}+ \lambda \,\Big [\frac{d \theta }{d X} \Big ]^2 - e^{- \,\xi \, X}\, \Big [N_{C}^2 + S_{h}\cdot \theta + N_{r}\cdot \theta ^3 \Big ]\times \theta + {\ddot{Q}}=0. \end{aligned}$$

We consider fin with adiabatic boundary conditions with finite length *L*. Further, at $x=0 \Rightarrow T=T_{b}$, and $\frac{d T}{d x}\Big \vert _{x=L}=0$ with dimensionless boundary conditions

11 $$\begin{aligned} \theta (0)= 1, \quad \quad \theta ' (1)= 0. \end{aligned}$$

## Entropy generation

Entropy equilibrium for every system go through any process using second-law of thermodynamics can be represented as^33^

12 $$\begin{aligned} {\ddot{S}}_{in}-{\ddot{S}}_{out} + {\ddot{S}}_{g}=\frac{d {\dot{S}}}{d t}, \end{aligned}$$

where entropy transfer rate by mass flowing at a rate of ${\dot{m}}$ and heat transfer rate ${\ddot{Q}}$ are ${\ddot{S}}_{mass} ={\dot{m}} {\dot{S}}$ and ${\ddot{S}}_{heat} ={\ddot{Q}}/T$. The entropy equilibrium can be represented on unit mass basis as

13 $$\begin{aligned} \sum _{i=1}^{n}\frac{{\ddot{Q}}}{T} \,+\,\sum _{i=1}^{n}\dot{m_{i}}{\dot{s}}_{i}- \sum _{i=1}^{n}\dot{m_{o}}{\dot{s}}_{o}\,+\, {\ddot{S}}_{g}= & {} \frac{d {\ddot{S}}}{dt}. \end{aligned}$$

For steady stat, $d {\dot{S}}/dt$ will be zero. By considering the output and input in control volume, it is possible to simplify the above equation in the form

14 $$\begin{aligned} \frac{d{\dot{q}}_{x}}{T(x)}\,-\,\frac{d{\dot{q}}_{x+dx}}{T(x+dx)}\,+\, {\dot{m}}({\dot{s}}_{i}-{\dot{s}}_{o})\,+\, {\ddot{S}}_{g}= & {} 0. \end{aligned}$$

Let fin made of in-compressible porous medium and air is an ideal gas, then an expression for $({\dot{s}}_{i}-{\dot{s}}_{o})$ can be expressed as^34^

15 $$\begin{aligned} ({\dot{s}}_{i}-{\dot{s}}_{o}) = -\int _{T_{a}}^{T(x)}\, \frac{C_{p}}{T} \,dT = -C_{p}ln\frac{T}{T_{a}}. \end{aligned}$$

Further, let $T(x+dx)-T(x)\approx 0$ and putting Eq. (15) in Eq. (14), we obtain

16 $$\begin{aligned} {\ddot{S}}_{g}= & {} {\dot{m}}C_{p}ln\frac{T}{T_{a}}\,+\,\frac{1}{T} \,\frac{\partial {\dot{q}}_{x}}{\partial x}dx. \end{aligned}$$

Putting $q\,=\,-\,k_{eff}\,A \,dT/dx$ in Eq. (16), we obtain

17 $$\begin{aligned} S_{g}^{''} = \frac{{\ddot{S}}_{g}}{W\,t \,dx} = -\,k_{eff}\,A \,\frac{\partial ^2 
T}{\partial x^2}\, + \, \frac{\rho \, C_{p}\, g\, K\, \beta }{t\, \nu }ln\frac{T}{T_{a}}(T\,-\,T_{a}), \end{aligned}$$

where $S_{g}^{''}$ represent entropy generation of the perforated fin depends on the physical and thermal characteristics of air and temperature profile within the fin. Using the dimensionless parameters, we obtain

18 $$\begin{aligned} \frac{S_{g}^{''}\,\times \,t^2}{k_{f}}= & {} D_{a}\,R_{a} \times \ln (1\, +\,T_{\gamma }\, \times \, \theta )\,\times \theta -k_{r}\,(t/L)^2\,\Big [\theta \, +\,(T_{\gamma }-1)^{-1}\Big ]^{-1}\frac{\partial ^2 \theta }{\partial X^2}. \end{aligned}$$

Putting $S_{h}=\frac{D_{a}\, R_{a}(L/t)^2}{K_{r}}$ in Eq. (18), we obtain

19 $$\begin{aligned} \frac{S_{g}^{''}\,\times \,L^2}{k_{eff}}= & {} S_{h} \times \ln (1\, +\,T_{\gamma }\, \times \, \theta )\,\times \theta - \Big [\theta \, +\,(T_{\gamma }-1)^{-1}\Big ]^{-1}\frac{\partial ^2 \theta }{\partial X^2}, \quad 0 \le X \le 1. \end{aligned}$$

The entropy generation number represented by $N_{s}$ is defined as $N_{s} =\frac{S_{g}^{''}\,\times \,L^2}{k_{eff}}$ gives

20 $$\begin{aligned} N_{s} = S_{h} \times \ln (1\, +\,T_{\gamma }\, \times \, \theta )\,\times \,\theta - \frac{e^{-\,\xi \, X}\, \Big [N_{C}^2 + S_{h}\cdot \theta + N_{r}\cdot \theta ^3 \Big ]\times \theta -\Big [\xi \,(1+\lambda \cdot \theta ) + P_{e}\Big ] \, \frac{d \theta }{d X} - \beta \,\Big [\frac{d \theta }{d X} \Big ]^2 - Q }{\Big [1 + \lambda \cdot \theta \Big ]\Big [\theta \, +\,(T_{\gamma }-1)^{-1}\Big ]}, \end{aligned}$$

where $T_{\gamma }$ is defined as

21 $$\begin{aligned} T_{\gamma }= & {} \frac{T_{b}}{T_{a}}. \end{aligned}$$

From Eq. (20), it is evident that $N_{S}$ is dependent on $S_{h}, N_{c}, N_{r}, P_{e}, Q, \lambda$, $\theta$ and temperature ratio $T_{\gamma }$. From Eq. (10), it is observed that dimensionless temperature $\theta$ also depends on $N_{c}, S_{h}, N_{r}, P_{e}, Q, \lambda$ and dimensionless length *X* of the exponential porous fin. Hence $N_{S}$ can be written as

22 $$\begin{aligned} N_{s}(X)= & {} g\,(T_{\gamma },\,S_{h},\,X). \end{aligned}$$

Moreover, the average entropy generation can be find in the whole fin by using the formula

23 $$\begin{aligned} S_A = \int _{0}^{1}\, N_{s}\,(X) \, dX = G\,(T_{\gamma },\,S_{h}). \end{aligned}$$

Equation (23) shows that $S_{A}$ depends on temperature ratio $T_{\gamma }$ and porosity parameter $S_{h}$.

### Numerical approach

The shooting approach is used to solve the model numerically. The boundary value problem (BVP) is divided into different initial value problems (IVPs) by the shooting approach. In general, we shoot trajectories in several directions until we identify one with the appropriate boundary value. The first step is to compute the Dirichlet BVP for a second-order linear differential equation

24 $$\begin{aligned} \frac{d^2 Y}{d x^2}=f(x) \frac{d Y}{d x}+g(x)\,Y+h(x) \quad \text {subject to}\quad Y(c)= \gamma ,\quad Y(d)=\delta , \end{aligned}$$

over an interval [c, d]. In this case, the solution to BVP is typically given by a linear combination of the functions ${\mu }(x)$ and ${\psi }(x)$, which are solutions to IVPs

25 $$\begin{aligned} Y(t)={\mu }(t)+\frac{\delta -{\mu }(d)}{\psi (d)}\psi (t), \end{aligned}$$

where ${\mu }(t)$ is a solution to IVP

26 $$\begin{aligned} \frac{d^2 {\mu }}{d t^2}=f(t)\frac{d {\mu }}{d t}+g(t)\,{\mu } +h(t), \quad \text {subject to}\quad {\mu }(c)=\gamma , \frac{d \mu (c)}{d t}=0, \end{aligned}$$

and ${\psi }(t)$ is a solution to another initial value problem

27 $$\begin{aligned} \frac{d^2 {\psi }}{d t^2}=f(t)\frac{d {\psi }}{d t}+g(t)\,{\psi } +h(t), \quad \text {subject to}\quad {\psi }(c)=\gamma , \frac{d \psi (c)}{d t}=0. \end{aligned}$$

## Numerical results and discussion

Here, we study numerically entropy generation inside growing exponential, decay exponential, and rectangular porous fin and compare results to specify suitable geometry for practical application of fin. We have proposed dimensionless equation for evaluation of entropy generation. The equations are solved numerically by Runge–Kutta method of order 4 (RK4). In moving exponential porous fin, the model equation is approximated by assuming radiation–conduction number $N_{r} = 0.4$, convection–conduction number $N_{c}=0.5$, Peclet number $P_{e}=0.4$, internal heat generation $Q=0.3$ and various values of $S_{h}$ and $T_{\gamma }$. First, we consider Eq. (10) to study the temperature distribution. Using the value of non-dimensional temperature $\theta$ calculated in Eq. (10), entropy generation number and average entropy for different fin geometries are calculated from Eqs. (20) and (23).

**Figure 2:**
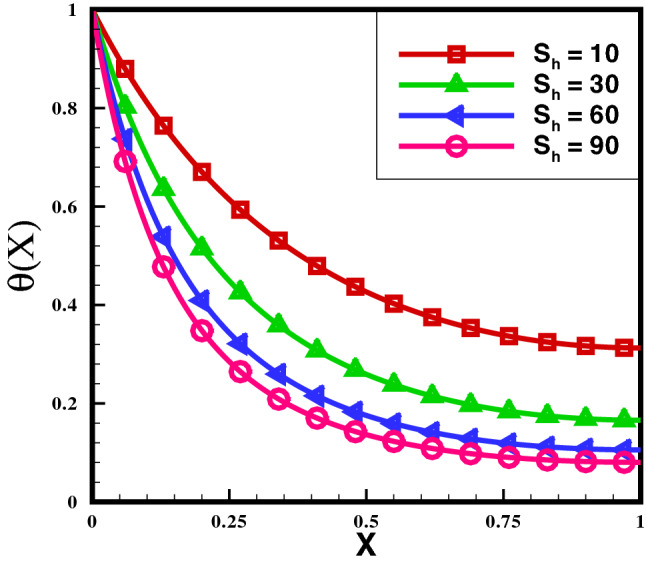
Effect of $S_{h}$ on temperature distribution.

The variance of temperature profile over the length of exponential porous fins is shown in Fig. 2. The results reveal that temperature distribution decreasing by increasing the numerical value of porosity. It is discovered that temperature profile of the solid fin is greater than that of the fin with higher porosity, because the high porosity decrease the effective heat conductivity of the fin due to the absence of solid materials. Simultaneously, convective heat transmission increases as more fluid passes through pores.

**Figure 3:**
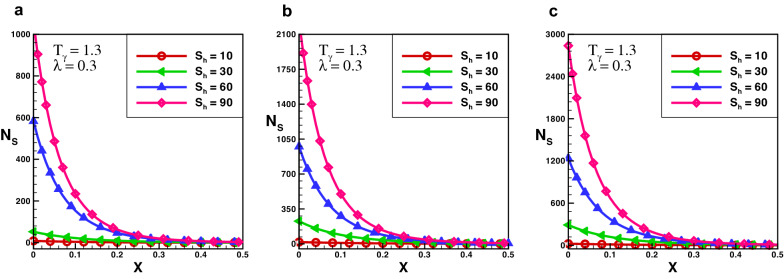
Entropy generation number in rectangular porous fin for $\lambda = 0.3$.

**Figure 4:**
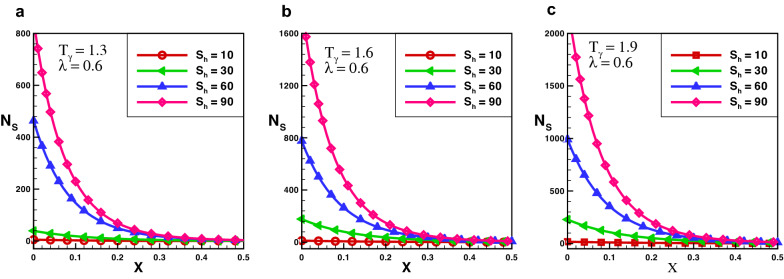
Entropy generation number in rectangular porous fin for $\lambda = 0.6$.

**Figure 5:**
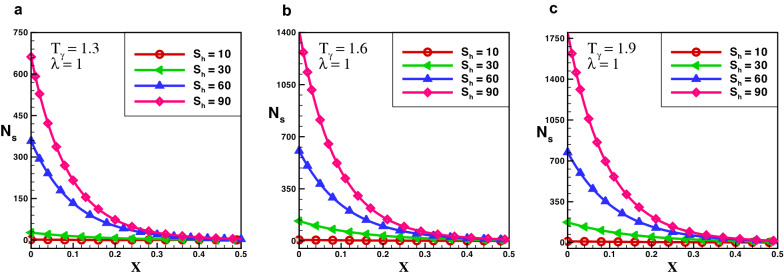
Entropy generation number in rectangular porous fin for $\lambda =1$.

The effect of entropy production for a porous rectangular fin with insulated tip for different values of $T_{\gamma }$ and $S_{h}$ are shown in Figs.  3, 4 and 5. From the figures, one can see that at specific values of $T_{\gamma }$ and $S_{h}$, the entropy generation is maximum at the fin’s base. It is evident that, particles of the material move faster when temperature increases around the fin’s base which increase the entropy production number. When temperature drops throughout the length of a fin, the measure of entropy production drops suddenly and the length of perforated fin increases and become negligible at fin’s tip. The particles of the material gain kinetic energy when the temperature ratio rises. The molecules that move faster at higher temperatures have more disorder than particles that move slowly at lower temperatures. The results also reveals that the influence of $T_{\gamma }$ is lower than that of $S_{h}$ as the porosity parameter attempts to oppose fluid flow and hence increases the overall entropy generation rate. By increasing the value of $S_{h}$ there is a large difference in entropy production which decreases as length increases, and for $X > 0.25$ the difference in entropy production is negligible. When the Rayleigh number is high, due to the enhanced buoyancy force and convection heat transfer, the fluid friction is greater which cause the entropy generation number $N_{s}$ to rise. Further, when the Darcy number grows, so does the entropy generation number. Thermal mixing is poor at lower Darcy numbers $(D_{a})$, and heat transfer irreversibility dominates overall entropy generation. It is also clear from these figures that thermal conductivity and entropy creation are inversely related. By increasing thermal conductivity parameter entropy generation will drop-down and vice versa. It conclude that, when thermal conductivity increases temperature distribution will increase, and as a result entropy generation decreases.

**Figure 6:**
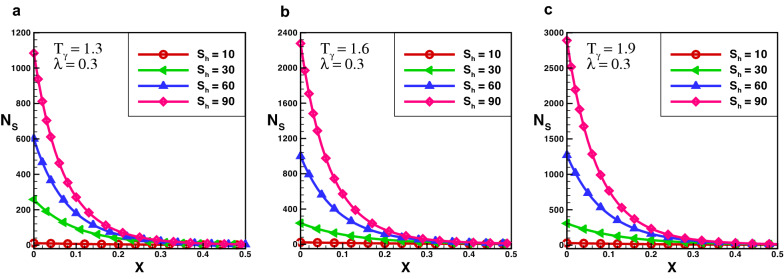
Entropy generation number in decay exponential porous fin for $\lambda = 0.3$.

**Figure 7:**
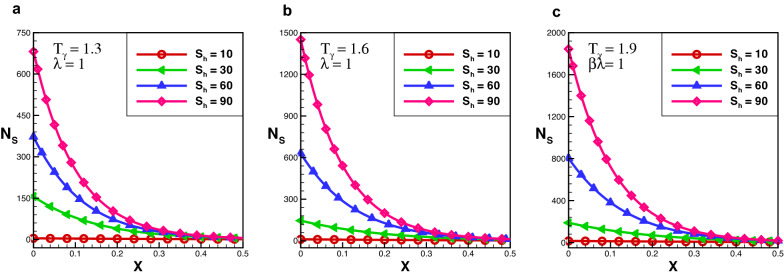
Entropy generation number in decay exponential porous fin for $\lambda = 1$.

The effect of entropy production for a decay exponential porous fin for a variety of values of $T_{\gamma }$ and $S_{h}$ are presented in Figs. 6 and 7. In the case of decay exponential fin, we observed that entropy generation is maximum at base of the fin and decreasing along fin’s length. The results indicate that there is a significant increase in entropy production number for several values of $S_{h}$ and this difference dropped sharply as the length of fin increases. By comparing the results in Figs. 3, 4 and 5 with Figs. 6 and 7 one can see that entropy production in the decay exponential fin is higher than rectangular fin. This is due to availability of more space for conduction of heat at base in case of decay exponential fin as a result molecules will obtain maximum kinetic energy and entropy generation will be maximum.

**Figure 8:**
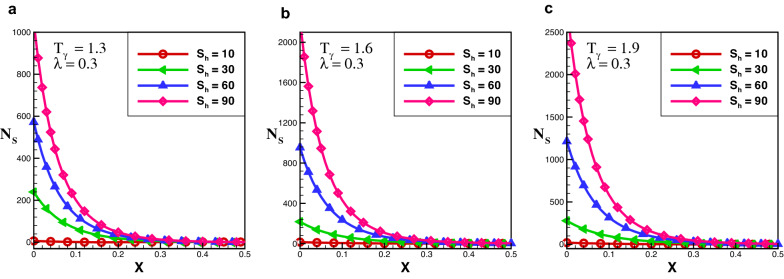
Entropy generation number in growing exponential porous fin for $\lambda = 0.3$.

**Figure 9:**
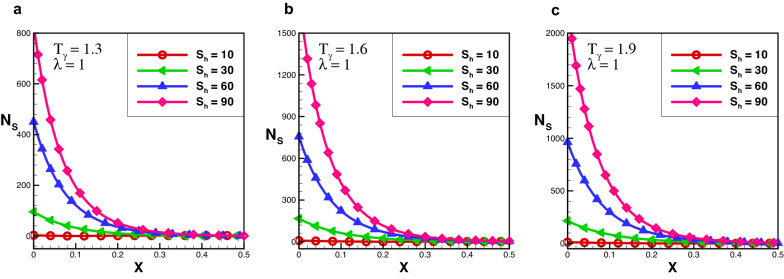
Entropy generation number in growing exponential porous fin for $\lambda = 1$.

The effect of entropy production for a growing exponential porous fin for a variety of values of $S_{h}$ and $T_{\gamma }$ are demonstrated in Figs. 8 and 9. One can see that in the case of growing exponential fin entropy generation number is maximum at fin’s base for rectangular and decay exponential. By comparing the results of entropy generation number in the case of rectangular, decay, and growing exponential fins it is observed that entropy production number in the decay exponential fins is higher than that of rectangular fin while entropy production number of the rectangular fin is lower than growing exponential fin. This is because more space is available for conduction of heat to the fin at the base in the decay exponential fin as a result entropy generation will be maximum.

Furthermore, the outcomes also show the effect of $T_{\gamma }$ and $S_{h}$ on $N_{s}$. It is obsrved that at particular value of $S_{h}$ by increasing the temperature ratio, entropy generation number increases. It is Also noted that, $S_{h}$ has a significant role in entropy generation at fin’s base, because for larger value of $S_{h}$, entropy generation increases exponentially, but this difference becomes smaller as fin length and become negligible at the tip of the fin. Entropy generation decreases as the length of the fin increases because temperature along the fin decreases. The effect of variable thermal conductivity parameter $\lambda$ is also analyzed and observed that entropy generation decrease as thermal conductivity increase for all cases. With the increase of thermal conductivity parameter temperature dissipation increases due to which kinetic energy of molecules decreases and as a result entropy generation drops-down.

**Figure 10:**
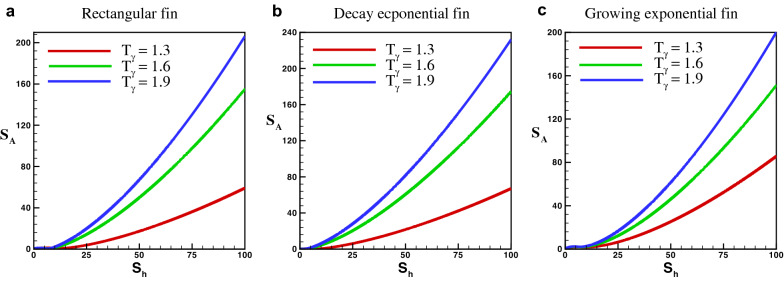
Average entropy for various fins geometries.

The effects for an average entropy of rectangular, decay and growing exponential fins with an adiabatic boundary condition for $\lambda = 1$ are presented in Fig. 10a–c, respectively. The results show that both $S_{h}$ and $T_{\gamma }$ are directly related to the average entropy of the perforated fins. The result also demonstrates that the influence of porosity parameter on the entropy production is larger as compared to $T_{\gamma }$. It is observed that with an increase in the value of $S_{h}$ from 1 to 80 average entropy generation increase about 14 times. However, increasing $T_{\gamma }$ from 1.1 to 1.9 an increase of about $30\%$ is observed. Hence, it is observed that the influence of $S_{h}$ and $T_{\gamma }$ on the average entropy in the adiabatic tip decay exponential porous fin is larger than that of the rectangular porous fin, which is higher than growing exponential porous fin. It is also observed that as the thermal conductivity increases entropy generation decreases.

## Conclusion

We have studied entropy generation in a variety of porous fins having temperature dependent thermal conductivity together with convection, radiation and internal heat generation. It is observed that entropy production number $N_{S}$ depends on porosity parameter, dimensionless temperature distribution and temperature ratio. The entropy generation obtained maximum at base of fin for specific value of $T_{\gamma }$ and $S_{h}$. The porosity parameter has a significant effect on entropy production around the fin’s base, but this effect reduces as we go nearer to the fin’s tip. Moreover, The influence of the porosity parameter on the entropy production number obtained is greater than that of the temperature ratio. The difference among the entropy production number for various values of $S_{h}$ and $T_{\gamma }$ is minimized as one approaches the fin’s tip. In various types of porous fins, boosting both $S_{h}$ and $T_{\gamma }$ has a direct influence on increasing the mean entropy production number. Hence, from the comparison, it is concluded that entropy generation in decay exponential fin is higher than growing exponential fin which is higher than rectangular fin. This means that, in the options of the porous fins, rectangular fin is one of the better choice.

## Abbreviations

*h*: Convection heat transfer coefficient $({\text {W}}/{\text {m}}^2\, {\text {K}})$
*W*: width of the fin (m)
$N_{r}$: Dimensionless radiation parameter
*g*: Gravitational acceleration (m/s$$^{2}$$)
*x*: Direction along x-axis (m)
$P_{e}$: Peclet number, dimensionless
$$T_{a}$$: Ambient temperature (K)
$T_{s}$: Dimensional surface temperature (K)
${\ddot{Q}}$: Dimensionless internal heat generation/absorption parameter
*K*: Porous fins permeability (Darcy)
$\tau _{b}$: Thickness of the fin’s base (m)
$k_{eff}$: Effective thermal conductivity (W/m K)
$k_{f}$: Air thermal conductivity (W/m K)
$${\ddot{S}}_{g}$$: Porosity parameter
$\theta$: Dimensionless local temperature
$\lambda$: Thermal diffusivity of air (m$^2$/s)
$\theta _{b}$: Dimensionless base temperature
$$\varepsilon$$: Surface emissivity
$\xi ^*$: Dimensional fin shape parameter
$$\eta$$: Fin efficiency
$\varphi$: Porosity of the fin
$\beta$: Volume expansion coefficient $({\text {K}}^{-1})$
$S_A$: Average entropy generation $({\text {W}}/{\text {m}}^3\,{\text {K}})$
$S_{g}^{''}$: Entropy generation of porous fin $({\text {W}}/{\text {m}}^3\,{\text {K}})$
*A*: Area of fin’s surface (m$^2$)
*P*: fins perimeter (m)
*U*: Speed of moving fin (m/s)
*X*: Dimensionless coordinate
*L*: Fins length (m)
$N_{c}$: Dimensionless convection parameter
$$T_{b}$$: Base temperature (K)
*T*: Dimensional fin temperature (K)
${\dot{q}}$: Internal heat generation (W)
${\dot{m}}$: Mass flow rate (kg/s)
$C_{p}$: Specific heat of the material (J/kg K)
$$D_{a}$$: Darcy number (K/t$$^2$$)
$$R_{a}$$: Rayleigh number $$\Big (g\,\beta \,t^3\,(T_{b}-T_{a})/\lambda \,\nu \Big )$$
$K_{s}$: Solid thermal conductivity (W/m K)
$k_{r}$: Thermal conductivity ratio $(k_{eff}/k_{s})$
$\tau _{b}$: Semi fin thickness m
$\theta _{a}$: Dimensionless ambient temperature
$\theta _{s}$: Dimensionless surface temperature
$\rho$: density of material (kg/m$^3$)
$\xi$: Dimensionless fin shape parameter
$\sigma$: Stefane-Boltzmann constant $({\text {W}}/{\text {m}}^2 \; {\text {K}}^4)$
$\nu$: Kinematic viscosity of air $({\text {m}}^2/{\text {s}})$
$N_{S}$: Entropy generation number
*V*(*x*): Velocity of buoyancy flow at point *x* $({\text {m}}/{\text {s}}^2)$
